# Habitat selection by a predator of rodent pests is resilient to wildfire in a vineyard agroecosystem

**DOI:** 10.1002/ece3.8416

**Published:** 2021-12-14

**Authors:** Allison E. Huysman, Matthew D. Johnson

**Affiliations:** ^1^ Department of Wildlife Humboldt State University Arcata California USA

**Keywords:** Barn Owl, ecosystem services, resource utilization function, vineyard, wildfire

## Abstract

Conservation of uncultivated habitats can increase the potential for ecosystem services in agroecosystems, but these lands are also susceptible to wildfires in the arid western United States. In Napa Valley, California, abundant rodent pests and an interest in integrated pest management have led wine producers to use nest boxes to attract Barn Owls (*Tyto furcata*) to winegrape vineyards. The viability of this practice as a method to control rodent pests depends heavily on the amount of hunting effort that Barn Owls expend in vineyards, which is known to be influenced by the amount of uncultivated land cover types surrounding the nest box. Wildfires burned nearly 60,000 ha of mainly urban and uncultivated lands surrounding Napa Valley in 2017, altering Barn Owl habitats. We compared GPS tracking data from 32 Barn Owls nesting in 24 individual nest boxes before and after the fires to analyze their hunting habitat selection. Owls with burned areas available to them after the fires had home ranges that shifted toward the fires, but selection was not strongly associated with burned areas. Though there was some spatial use of burned areas, selection of land cover types was similar for birds before and after the fires and in burned and unburned areas. The strongest selection was for areas closest to the nest box, and most recorded locations were in grassland, though selection indicated that owls used land cover types in proportion to their availability. Overall, habitat selection was resilient to changes caused by wildfires. These results are important for farmers who use nest boxes as a means of rodent control, which may be affected after dramatic disturbance events, especially as wildfires increase in the western United States.

## Graphical abstract

Uncultivated habitats in Napa Valley, California can help attract Barn Owls to nest boxes in agroecosystems, though these habitats are also at risk of wildfire. After several large wildfires in the region, GPS tracking of Barn Owls revealed that their habitat selection is resilient to fire, though they show some spatial preference for fire edges. This is important for farmers who use nest boxes as a means of rodent control as wildfires increase in the western United States.

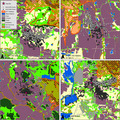

## 1 INTRODUCTION

Recent literature has focused on the link between pest control by natural enemies and the composition of the surrounding landscape (Lindell et al., 2018). Conserving native or uncultivated habitats and maintaining landscape heterogeneity can increase the potential for ecosystem services, particularly pest control (Garibaldi et al., 2020; Grass et al., 2019; Kremen & Merenlender, 2018). The composition of the landscape surrounding agricultural areas is critical when the agents providing pest control are mobile and rely on resources beyond those provided by cultivated habitats. Much of the literature surrounding the relationship between landscape composition and mobile agents that provide ecosystem services has focused on pollinators (Kremen et al., 2007) and insect predators (Boesing et al., 2017; Veres et al., 2013), but this theory also applies to organisms that provide vertebrate pest control.

Among the most popular mobile agents of vertebrate pest control are Barn Owls (*Tyto alba* and *Tyto furcata*), which are used in integrated pest management systems worldwide (Labuschagne et al., 2016). There is increasing interest in maximizing pest control by Barn Owls, which prey upon pest species including rats, gophers, voles, and mice (Labuschagne et al., 2016). They make an excellent candidate species for pest control, as they readily use human‐made nest boxes, will nest at high densities if food is abundant, produce large numbers of young throughout the breeding season (Barn Owl Trust, 2012; Roulin, 2020; Taylor, 1994), and consume large numbers of rodent pests each year (St. George & Johnson, 2021). Furthermore, their adaptability to different climates and landscapes, and their opportunistic prey selection of locally common rodent species have helped them achieve a global distribution and succeed in human‐dominated landscapes (Roulin, 2020). Because of these qualities, Barn Owl nest boxes are often placed in agricultural areas with the hopes of controlling rodent damage, but their ability to deliver pest control depends on how much owls hunt in cultivated areas (Johnson et al., 2018).

In California, American Barn Owls (*Tyto furcata*) are important predators of pests such as Botta's pocket gophers (*Thomomys bottae*) and voles (*Mictrotus* spp; St. George & Johnson, 2021), though the efficacy of Barn Owls as a pest control agent remains unresolved (Kross & Baldwin, 2016; Labuschagne et al., 2016). Recent research has shown that Barn Owls in Napa Valley, California, preferentially use nest boxes near uncultivated lands, meaning land cover types such as grasslands, oak savannah, and riparian habitats (Wendt & Johnson, 2017) that are not intensively managed and were present in the landscape prior to European colonization. These are similar to “native” habitats as defined by Garibaldi et al. (2020), though in our system many of the grasslands are currently dominated by non‐native annual grasses. Barn owls show strong selection for grasslands (Huysman & Johnson, 2021; Wendt & Johnson, 2017), and when more of this habitat is available, Barn Owls spend less time hunting in vineyards (Castañeda et al., 2021). In addition to hunting behavior, the landscape surrounding Barn Owl nest sites in various parts of the world has been shown to affect nesting (Charter et al., 2010; Hindmarch et al., 2014), exposure to anticoagulant rodenticides (Hindmarch et al., 2017), and nest box occupancy (Hindmarch et al., 2012; Wendt & Johnson, 2017). Thus, the composition of vineyards and uncultivated land cover types throughout the landscape has consequences for the potential of Barn Owls to both nest and hunt in vineyards, therefore affecting their potential ability to control pests.

Highly heterogeneous Mediterranean habitats, such as those in California, are prone to wildfires, which adds additional landscape complexity that wildlife may respond to. The composition of uncultivated land, the preferred hunting habitat for Barn Owls in Napa Valley (Castañeda et al., 2021), was altered by large wildfires in the region in 2017. The Atlas, Nuns, and Tubbs fires burned nearly 60,000 ha around Napa Valley, primarily affecting uncultivated land cover types (Lapsley & Sumner, 2017). Landscape changes caused by these fires also have the potential to change vegetation structure and rodent communities on which Barn Owls depend. In the western United States, small mammals are likely to have increased populations in recently burned areas (Fitzgerald et al., 2001; Schwilk & Keeley, 1998), and in other recently burned Mediterranean climates, fires produce edge habitat and open areas that seem to be preferred by rodents (Haim & Izhaki, 1994; Torre & Díaz, 2004). If fires cause significant changes to rodent communities in the habitats that Barn Owls prefer, it is likely that Barn Owls would respond by hunting where prey is more available.

The response of both Barn Owls and rodents to these fires is likely to be affected by the natural fire regimes in the region and the current trend of increasing fire frequency and severity. Wildfires in the western United States are currently fueled by a changing climate and decades of fire suppression (Batllori et al., 2013). Furthermore, the conversion of native perennial grasses to non‐native annual grasses in much of California is increasing the availability of fine fuels and causing more frequent fires (Jurjavcic et al., 2002; Keeley & Brennan, 2012). In Napa Valley, like much of California, fire was previously a tool frequently used by Indigenous tribes to maintain an open landscape and prevent the likelihood of larger fires (Grossinger, 2012). Fires have been suppressed since colonial settlement and warming, earlier springs (Westerling et al., 2006), and fuel accumulation (Agee & Skinner, 2005; Westerling et al., 2003) have led to increased frequency and intensity of wildfires in the western United States since the 1980s. Wildlife in the western United States shows mixed responses to wildfire, though species that are mobile and use open land cover types generally are resilient in response to novel fire regimes (Fontaine & Kennedy, 2012; Jager et al., 2021; Jones et al., 2020). Most research to date has focused on wildlife response to forest wildfires (Jager et al., 2021), and it is unknown how novel fire regimes will alter agricultural landscapes, or if the history of frequent, low‐intensity fires in the region will mean that the environment and wildlife that inhabit it are resilient to the large fires that the region is experiencing today.

The objective of this study was to determine whether Barn Owl habitat use changed as a result of wildfire. Barn Owls are central‐place foragers that are more likely to hunt near the nest box (Taylor, 2009), and they preferentially choose to hunt in uncultivated land cover types, especially grasslands, when it is available (Castañeda et al., 2021). Wildfire can alter vegetation structure in these land cover types and affect habitat selection. For example, other central‐place foraging owl species have shown selection for low and moderate‐severity burned forests far from their territories (Bond et al., 2009; Eyes et al., 2017). This is likely because of increased rodent populations and easier hunting in burned areas where the tree canopy is made more open by fire. The selection of burned patches may also signify adaptation to historical fire regimes, as has been shown in Spotted Owls (*Strix occidentalis*; Jones et al., 2020). The global distribution of Barn Owls in many fire‐prone landscapes suggests that this species should be resilient to fire‐caused changes to the landscape. Given that wildfires alter Mediterranean landscapes by fragmenting dense habitat and creating edges (Parkins et al., 2018) that can promote rodent abundance (Fitzgerald et al., 2001; Haim & Izhaki, 1994; Schwilk & Keeley, 1998; Torre & Díaz, 2004) and that Barn Owls are central‐place foragers that prefer open habitat types (Castañeda et al., 2021; Séchaud et al., 2021), we hypothesized that Barn Owls would continue to select their preferred hunting habitats, that they would hunt near their nest box, and that they would show selection for intermediate levels of fire severity and proximity to the fire edge even when the fire is far from the nest box as has been shown in other central‐place foraging owl species (Bond et al., 2009; Eyes et al., 2017).

Using a design involving studying owls that did and did not experience significant fire in their home ranges both before and after a wildfire, we investigated whether Barn Owl habitat selection differed among nest boxes affected and unaffected by fire before and after a wildfire event. We addressed this question by examining Barn Owl land cover selection among nest boxes that were and were not affected by wildfire both spatially and temporally. We also look at the response of nest boxes near the fire in a post‐fire habitat selection analysis to understand how Barn Owls use a burned landscape. An understanding of how owls respond to wildfire is crucial for determining how resilient Barn Owl pest control is to disturbance and changes to landscape composition. Mediterranean climates such as Napa evolved with fire, though the whole western United States is experiencing increasing fire intensity and frequency due to fire suppression and changing climate conditions (Batllori et al., 2013; Westerling et al., 2006). The increased likelihood of intense fires could affect the ability of mobile predators to provide pest control throughout a disturbed landscape.

## 2 METHODS

### 2.1 Field methods

#### 2.1.1 Study area

This project took place on vineyards in Napa Valley, California, where 273 nest boxes have been monitored since 2015. Napa Valley is ~48 km long and 5–20 km wide and is characterized by a Mediterranean climate ideal for growing grapes (Napa Valley Vintners, 2017). Mixed oak woodlands and oak savannahs are spread throughout the region, with more oak‐grasslands in the south and more mixed oak scrub and conifer forests in the north (Napa County, 2010; Wendt & Johnson, 2017). The unique conditions in Napa County have created a wine industry that generates $3.7 billion in revenue each year and in combination with tourism, employment, and distribution, is estimated to have an annual impact of $50 billion on the American economy (Stonebridge, 2012).

**FIGURE 1:**
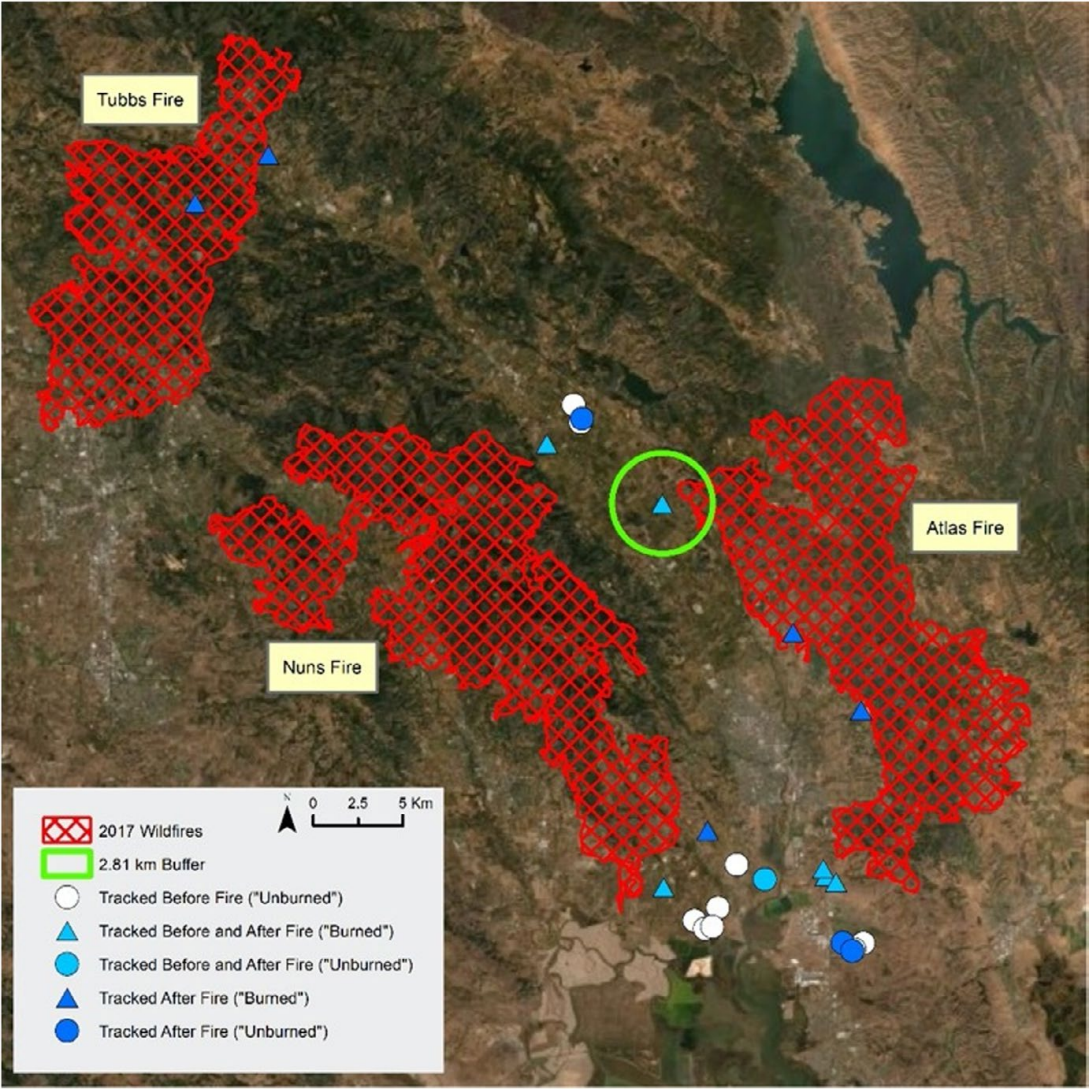
Map of Napa Valley, California, study area with nest boxes tracked by GPS telemetry and Atlas, Nuns, and Tubbs fires. Green circle represents 2.81 km buffer around nest box, the mean maximum distance traveled from the nest box for Barn Owls in this population. Triangles indicate nest boxes that were classified as “burned” based on 2.81 km buffer, and circles indicate nest boxes classified as “unburned.” No nest boxes that were classified as “burned” were tracked only before the fires. Service layer credits: Esri, DigitalGlobe, GeoEye, Earthstar Geographics, CNES/Airbus DS, USDA, USGS, AeroGRID, IGN, and the GIS User Community

While the Atlas, Nuns, and Tubbs fires of October, 2017 burned over 60,000 hectares surrounding Napa Valley (Cal Fire, 2017), few vineyards were burned (Lapsley & Sumner, 2017; Figure 1) due to their comparatively low fuel levels and more mesic conditions than surrounding land cover types. The fires primarily burned in urban areas, oak savannah, grassland, and other uncultivated land cover types (Lapsley & Sumner, 2017). These uncultivated areas were burned at a variety of burn severities within the fire perimeter, introducing variability in impacts to soils and vegetation, but this did not result in the conversion of any land cover type to another (California Department of Forestry & Fire Protection, 2017a).

#### 2.1.2 Study species

During the breeding season, when Barn Owls occupy nest boxes, the male and female alternate hunting depending on the stage of raising young. Taylor (1994) observed that the percentage of males that sit beside their females in the nest increases to reach a peak in the two weeks before laying and during laying. After this point, male and then female hunting increase in an effort to meet the varying metabolic requirements of young in the nest, with males doing most of the hunting and provisioning the young and the female in the first couple of weeks, followed by both adults hunting actively to provision the young in Weeks 3–10 (Bank et al., 2019; Durant & Handrich, 1998; Taylor, 1994). Adult male owls generally weigh 400–560 g and females weigh 420–700 g (Marti et al., 2005).

#### 2.1.3 GPS telemetry

We deployed Global Positioning System (GPS) tags on 15 Barn Owls throughout the breeding season in Napa Valley in 2018, adding to a sample of 17 owls which were tagged during the 2016 and 2017 breeding seasons (Castañeda et al., 2021). Selecting birds to tag depended on occupancy of boxes during tagging occasions, but when feasible, we tagged owls that had either no burned area or large amounts of burned area within their home range and prioritized next boxes and individuals previously tagged in 2016 (Castañeda et al., 2021). All birds tagged were females for consistency with Castañeda et al. (2021) and because females more reliably return to the nest box during the day than the males, which aided retrieval of GPS data and GPS tag recovery. Birds were tagged if there were young at least two weeks old in the nest, as laying and incubation are considered more sensitive stages of the nesting cycle (Meyrom et al., 2009) and because this period coincides with maximum hunting by adults to meet the metabolic requirements of nestlings (Martin et al., 2010; Naim et al., 2010; Séchaud et al., 2021).

Birds were trapped within the next box during the day. We first blocked the entrance to a box with a pillow, then climbed a ladder, and removed the owls through a door on the side or top of the box. The owls were placed in a pillowcase to remain calm until processing and then had their eyes covered with a cloth hood during banding and tagging. Each tagged bird was also given a unique U.S. Geological Survey aluminum band. Weight and morphological measurements including wing and bill length were also recorded for each bird. We confirmed the sex of each bird by the presence of a brood patch. When tagging and data collection were finished, we placed the birds back in the nest box and blocked the entrance for an extra five minutes to allow the birds to calm down before we left the site. Total handling time did not exceed 20 min per bird.

Global Positioning System tagging followed the protocol of a previous study on the same population (Castañeda et al., 2021), using the Uria 300 tags developed by Ecotone Telemetry (2015), which weigh 13.5 g each. Tags were attached to birds using a small harness created with a Teflon ribbon that does not interfere with the bird's movements (Humphrey & Avery, 2014). We programmed our units to record a location every two minutes after sunset and before sunrise each night. Castañeda et al. (2021) collected locations every one minute, so we subsampled the data from those individuals to every two minutes for analytical purposes.

Data from deployed tags were downloaded remotely though a handheld base station left at the nest box. We collected data on each bird for 14 to 21 days, the approximate battery life expectancy with our programmed location frequency, and then attempted to recover the tag so it could be deployed on another owl. If the female was still roosting diurnally in the nest box, we re‐trapped the female within this box as described above. When that was not possible because the female was no longer roosting in the nest box, we used a custom trap attached to the nest box (as described by M. Charter, Personal Comm.) that allowed us to recapture adults at night when they came to deliver prey to nestlings. After recovery of the tag, we removed the trap and returned the adult to the nest box as during diurnal handling.

### 2.2 Statistical analyses

#### 2.2.1 Treatment groups

This study focused on owls occupying 24 individual nest boxes, 9 of which were monitored before the Atlas, Nuns, and Tubbs fires of 2017, 7 monitored after the fires, and 8 monitored both before and after the fires (Figure 1). Though none of the boxes themselves in this study were burned, boxes within 2.81 km of the 2017 wildfires were classified as “burned” from an owl habitat perspective because this was the mean maximum distance moved by all GPS tracked individuals in this study.

We chose to group the nest boxes using a standard radius so that the groups would reflect what was available as hunting habitat for each nest box. We used utilization distributions to characterize home range (see Brownian Bridge Movement Model), but these inherently include some degree of habitat selection; therefore, a standard radius around each nest box may better depict the habitats available to hunting owls (Comfort et al., 2016). Given that owls may travel far from their nest to access burned areas (Bond et al., 2009; Eyes et al., 2017), a standard radius based on the distance that we know individuals in this population is capable of traveling from the next box better grouped individuals based on what habitat was available to them and enabled comparisons between years. The use of the 2.81 km radius meant that boxes further than this distance from the wildfire perimeter were classified as unburned. Thus, the 24 nest boxes were divided into four groups: pre‐fire unburned (*n* = 10), post‐fire unburned (*n* = 4), pre‐fire burned (*n* = 7), and post‐fire burned (*n* = 11), depending on the year(s) the box was studied and whether the nest box had burned habitat within an owl's hunting radius (Figure 1). These groupings enabled analytical comparisons, but it is important to note the limitations imposed by our opportunistic study design. Namely, it was not feasible for our entire sample to be the same owls both before and after fire in burned and unburned habitat.

We compared the proportion of land cover types used by the pre‐fire and post‐fire groups by calculating the alpha generalized correlation between the two groups in the R package *Compositional* (Tsagris et al., 2021). We also compared the proportion of land cover types available to each group by calculating the correlation coefficient of the land cover composition of the 2.81 km radius around the pre‐fire and post‐fire nest boxes and around the “burned” and “unburned” nest boxes. We log‐transformed the data before calculating the correlation to accommodate assumptions of normality.

#### 2.2.2 Brownian bridge movement model

We used the package *move* (Kranstauber et al., 2015) in program R version 3.5.1 (R Core Team, 2020) to build dynamic Brownian Bridge Movement Models (hereafter referred to as dBBMM) for all 32 tagged birds. We only used GPS locations that were collected more than 30 m from the nest box, the approximate range of the base station located at the nest box, to exclude locations when the bird was in or very near the box and to account for GPS error. The GPS transmitters were programmed to record three locations at a time, and we removed duplicate timestamps so that only the first of each timestamp was used. We removed duplicate locations rather than averaging because in many cases, the individual was moving, and an average of the locations could produce a location where the individual was never actually observed. After constructing the dBBMM, we cropped it to the 95% utilization distribution for further analysis.

#### 2.2.3 Resource utilization function

We used the package *ruf* (Handcock, 2011) in program R to build Resource Utilization Functions (RUF) as described by Marzluff et al. (2004), which allow the use of a utilization distribution as the response variable to estimate resource selection. An advantage of the RUF approach is that the utilization distribution (UD, in this case, dBBMM) is able to account for any spatial–temporal autocorrelation and minimizes errors associated with GPS fixes (Hooten et al., 2013; Kranstauber et al., 2012; Millspaugh et al., 2006). RUF models use a multiple regression approach to relate UD estimates to resources, where resulting coefficients indicate the importance of each resource to variation in the UD (Marzluff et al., 2004). Depending on when a bird was tracked, we used predictors in RUF models based on rasters for land cover type, soil burn severity, distance to fire edge, and distance to nest box.

Soil burn severity and fire edge data were obtained from Cal Fire (California Department of Forestry & Fire Protection, 2017a, 2017b, 2017c). The soil burn severity map is produced using a combination of Landsat satellite imagery‐derived Burned Area Reflectance Classification (BARC) maps and field observation, with a resulting raster product of four categories: very low/unburned, low, moderate, and high severity (California Department of Forestry & Fire Protection, 2017a, 2017b, 2017c). Because the severity categories were classified on an ordinal scale, we treated the burn categories as continuous (from least burned at 1 to most severely burned at 4) to determine which part of the severity scale was most highly selected. The land cover raster was created using a combination of remote sensing using NAIP (USDA, 2009) and LiDAR (NSF, 2013) and existing GIS layers (County of Napa, 2010; USDA & NASS, 2019) to classify land cover into seven categories at 4 m resolution: water/wetland, urban, vineyard, grassland, oak savannah, mixed forest, and riparian. Because land cover was represented as seven factors of a single categorical variable, we used deviation coding in all RUF models. For this reason, each land cover variable is presented and interpreted as a contrast against the mean effect of all land cover types in each model (Johnson et al., 2004).

Prior to analysis, the UD was rescaled for each individual to a range between 0 (lowest use) and 100 (highest use) based on the height of the UD at each grid cell (Kertson & Marzluff, 2011). We then added 0.01 to the rescaled UD values and log‐transformed them to meet assumptions of normality (Butler et al., 2020; Hooten et al., 2013). For each raster cell of the UD, we extracted the height of the UD and the corresponding values of each predictor variable for the RUF analysis.

For birds that were GPS tagged after the fires and had some burned area within a 2.81 km radius of their nest box (*n* = 11), we built a RUF model using the predictors: land cover, distance to nest box, distance to fire edge, soil burn severity, and soil burn severity squared, because we hypothesized that Barn Owls would select for an intermediate level of burn severity (represented mathematically by including a quadratic term), similar to other owl species (Bond et al., 2009; Eyes et al., 2017). We averaged the coefficients from each predictor for all birds and used Marzluff et al.’s (2004) recommendation for calculating standard deviation and confidence intervals from averaged coefficients.

To determine whether the fires affected habitat selection, we constructed RUF models for all 32 birds using the land cover and distance to nest box rasters as predictors. We also used the centroid of the UD, which we defined as the location with the highest probability of utilization, to assess spatial differences in each individual's home range. We ran 2‐way ANOVAs with distance between centroid and the nest box and distance between centroid and the fire perimeter as the response variables, and burn group (burn or no burn) and year (pre‐fire or post‐fire) as the grouping variables, with additive and interactive models for these terms. This design included individual owls that did and did not experience significant fire both before and after the naturally occurring fires, but it is not a strict Before‐After‐Control‐Impact (BACI) experimental design because subjects were obviously not chosen randomly; rather, they were made possible by the availability of previous data (Castañeda et al., 2021) and the distribution of the wildfires and occupied nest boxes.

## 3 RESULTS

**FIGURE 2:**
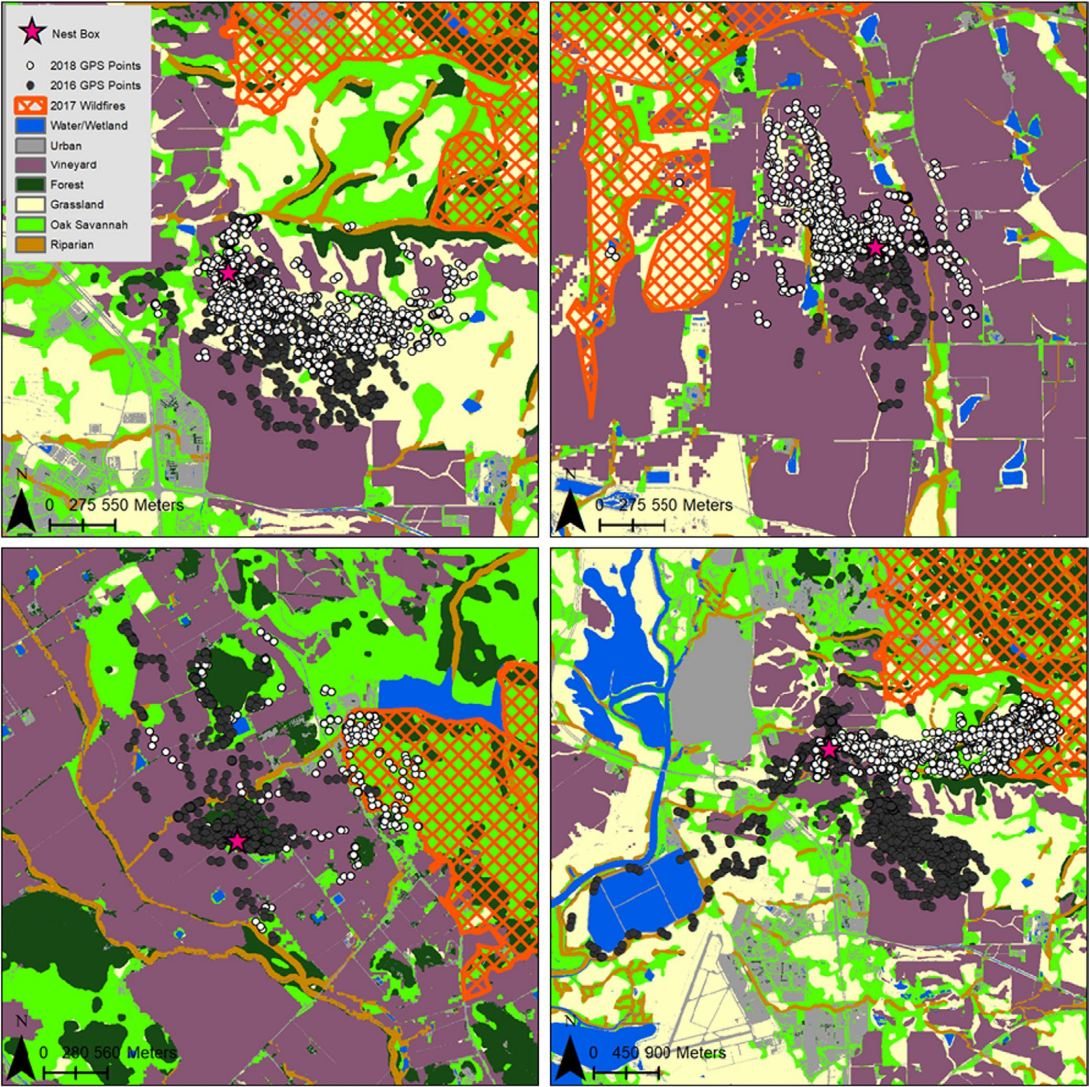
Map of Napa Valley land cover classification and GPS telemetry points collected on owls tracked before the fires (2016) and after the fires (2018), with area burned. The top two nest boxes were the same individuals tracked in the depicted nest box before and after the fires (one individual per panel, tracked in different years, with year depicted by color of points). The bottom two nest boxes were different individuals tracked in the depicted nest box before and after the fires (two individuals per panel, with individual and year depicted by color of points)

**FIGURE 3:**
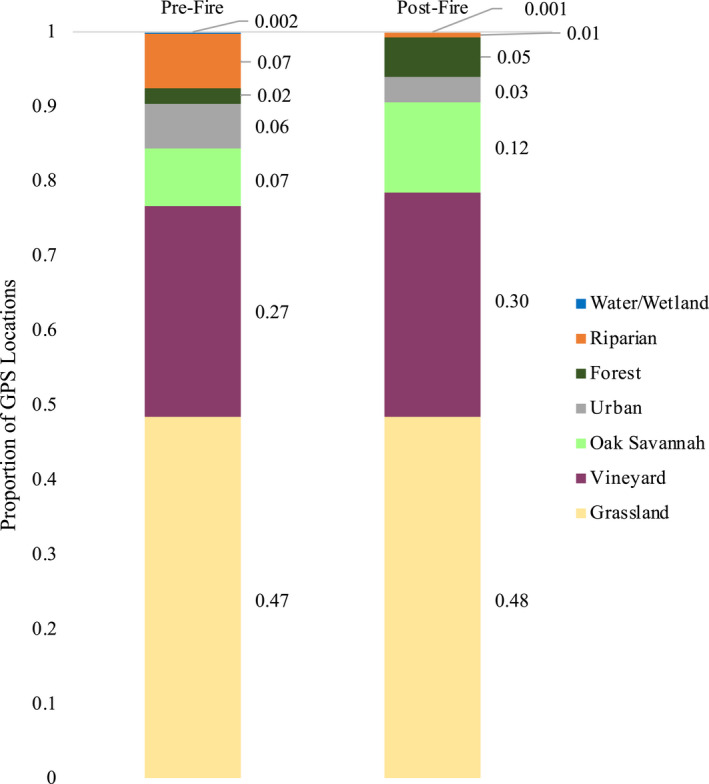
Proportion of GPS locations for Barn Owls in seven land cover types before wildfires (*n* = 17) and after wildfires (*n* = 15)

Between 2016 and 2018, GPS data were collected on 32 birds. After removing locations within 30 m of the nest box and duplicate timestamps, the mean number of locations collected per individual in a year was 851 (range 114–1876). Throughout the four nest box groups (ten pre‐fire unburned, seven pre‐fire burned, four post‐fire unburned, and eleven post‐fire burned), eight nest boxes and three individuals were studied both before and after the fires (example of data from four nest boxes in Figure 2). GPS locations pooled before and after the fires were composed of approximately 48% in grassland, 29% in vineyard, 10% in oak savannah, and less than 5% in each water, urban, mixed forest, and riparian land cover types. There was a similar proportion of locations in each land cover type for nest boxes studied pre‐fire and post‐fire (Figure 3). The correlation coefficient of GPS locations pre‐ and post‐fire was 0.978, indicating a high level of similarity in land cover composition used by each group. The composition of land cover types available to each owl (within the 2.81 km radius of the nest box) was also highly similar, with a correlation coefficient of 0.887 between the pre‐fire and post‐fire nest boxes and 0.819 between the “burned” and “unburned” nest boxes. The mean percent of burned area within the 2.81 km radius of “burned” nest boxes was 28.83% (± 8.97 SE, range 7.11–99.1).

After constructing a dBBMM for each individual, the average home range was 37.31 km^2^ (± 6.62 SE). This corresponded to an average of 2,331,908 UD and predictor values for each individual at 4 × 4 m resolution for use in the RUF analysis.

**FIGURE 4:**
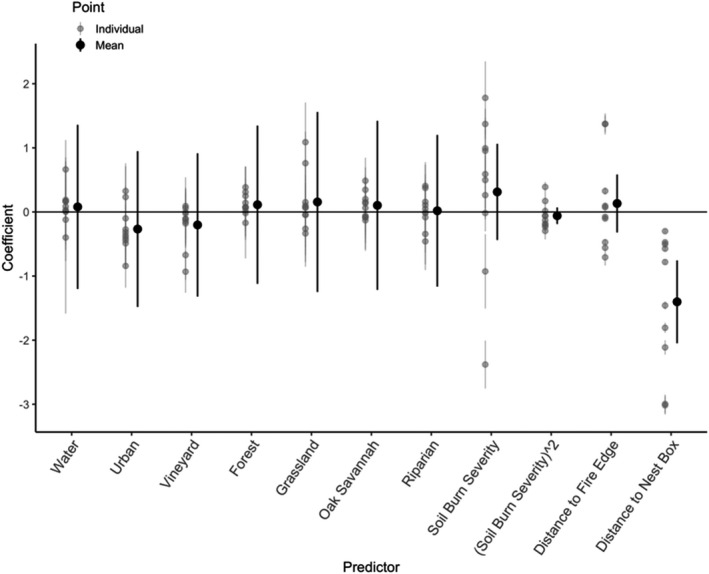
Mean selection coefficients and 95% confidence interval for each predictor variable in resource utilization function model, UD = land cover + distanceToNestBox + soilBurnSeverity + (soilBurnSeverity)^2^ + distanceToFireEdge, calculated for 10 birds that were GPS tagged near burned areas after the fires. Black dots show mean of all coefficients, and gray dots show coefficients for each individual

We evaluated one RUF model for the group of 11 birds that were GPS‐tagged near burned areas after the fires, with the formula UD = land cover +distanceToNestBox + soilBurnSeverity + (soilBurnSeverity)^2^ + distanceToFireEdge (Figure 4). This model converged for 10 of the 11 birds; one bird did not have enough variation in soil burn severity for the model to converge. At the population level, the mean coefficient for all land over types had confidence intervals that overlapped zero, indicating variability among individuals and that each land cover type was used in proportion to its availability. At the individual level, grassland and riparian were the land cover types with the most individuals with positive selection and confidence intervals that did not overlap zero. Selection was negative for distance to nest box, meaning intensity of utilization was negatively associated with this variable, and the confidence interval did not overlap zero at both the population level and for every individual. At the population level, confidence intervals overlapped zero for fire edge, soil burn severity, and soil burn severity squared, indicating variability in selection. At the individual level, 8 individuals had confidence intervals that did not overlap zero for distance to fire edge, 5 of which had negative selection, indicating a preference to be closer to the fire edge. Eight individuals also had confidence intervals that did not overlap zero for soil burn severity, 6 of which had positive selection. Seven individuals had confidence intervals that did not overlap zero for soil burn severity squared, 5 of which were negative, which coupled with positive selection for soil burn severity indicates a selection for intermediate levels of severity.

**FIGURE 5:**
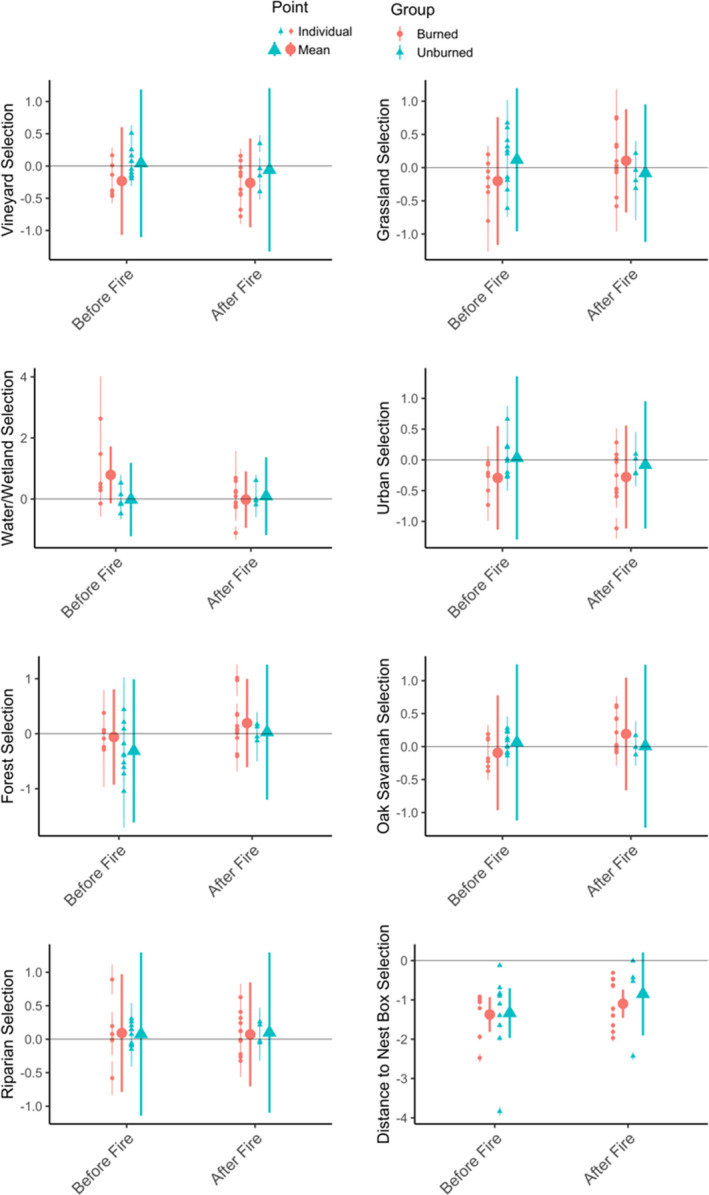
Mean and individual selection coefficients with 95% confidence intervals for each predictor variable in resource utilization function model, UD = vineyard + forest + grassland + riparian + oakSavannah + urban + water + distanceToNestBox, calculated for all 32 birds. Colors and symbols indicate whether birds were tracked before or after the 2017 fires, and whether or not their hunting range was near burned habitat

For all 32 tagged birds, the average coefficients for the seven land cover types did not show consistent differences between groups. The confidence interval for each land cover type for each group overlapped zero, indicating both variation among individuals and that at the population level, land cover types were used in proportion to their availability (Figure 5). Distance to nest box was strongly negative at the population and individual levels, with confidence intervals that did not overlap zero for all groups. Though the pattern was not strong at the population level, 23 individuals had confidence intervals that did not overlap zero for vineyard, 17 of which had negative selection. Grassland similarly had 20 individuals with confidence intervals that did not overlap zero, 10 of which had positive selection and 10 of which had negative selection. In the pre‐fire unburned group, grassland had the most individuals with positive selection (*n* = 5) and confidence intervals that did not overlap zero and forest had the most with negative selection (*n* = 4). In the pre‐fire burned group, water/wetland had the most individuals with positive selection (*n* = 5) and vineyard had the most individuals with negative selection (*n* = 5). In the post‐fire burned group, oak savannah was the land cover type most positively selected (*n* = 5), while again vineyard was the most negatively selected (*n* = 7). The post‐fire unburned group had weaker effects at both the individual and population levels, with no land cover types being selected or avoided more than others.

The centroid of the Brownian Bridge home range differed among groups, generally suggesting birds would travel farther to hunt near burned areas after the fires. The mean distance between the centroid and the nest box for all pre‐fire nest boxes (*n* = 17) was 223 m (± 110 SE) and for all post‐fire nest boxes (*n* = 15) was 619 m (± 264). The mean distance between centroids for the same nest box in different years (for nest boxes studied both before and after the fire) was 643 m (± 318). Though the distance between the centroid and the nest box differed among groups, the results of a two‐way ANOVA testing whether year, burn (and interactive or additive models), or a null model best explained these differences, the null model had the most support (Appendix 1). However, all other models were within 4 ∆AICc, meaning there was a high level of model uncertainty.

The mean distance between the centroid and the fire edge also differed between groups. The mean distance between the centroid and the (future) fire edge for all pre‐fire birds was 2865 m (± 361) and for all post‐fire birds was 1692 m (± 344). For just the nest boxes within 2.81 km of the fire edge (those classified as “burned”), the mean distance between the centroid and the fire edge for pre‐fire birds was 1180 m (± 121) and for post‐fire birds was 1051 m (± 211). In the two‐way ANOVA to test whether year, burn (and interactive or additive) models best explained these differences among groups, the burn model had the most support (Appendix 1). The additive and interactive models (burn + year and burn * year) were within 2 ∆AICc of the burn model, while the year and null models had very little support (∆AICc > 55).

## 4 DISCUSSION

Barn Owl habitat selection was resilient to landscape changes caused by wildfires. Specifically, the owls showed some spatial selection for burned areas by using areas near the fire (Figure 2, Appendix 1), but overall this did not change the land cover types they selected (Figure 3). Though the effect was weak, Barn Owls with access to burned areas after fire showed some selection for low to intermediate levels of burn severity, which is also consistent with studies of other owl species post‐fire (Bond et al., 2009; Eyes et al., 2017). The selection that some individuals showed for burned edges is consistent with edge effects created by fire (Parkins et al., 2018) which cause small mammals to spend time where burned and unburned areas meet (Haim & Izhaki, 1994; Schwilk & Keeley, 1998). Ultimately, this sample of Barn Owls showed some spatial preference for burned areas, with utilization distributions shifting in the direction of the fire, but this had minimal effects on their potential for pest control because it did not substantively change their selection of land cover types. Though RUF coefficients show variation in selection, the distribution of locations among land cover types for all birds, regardless of year and proximity to burn, showed a marked preference by the owls for uncultivated land and a similar use of land cover categories overall.

Previous research has shown that the same land cover variables that are associated with nest box occupancy are also the land cover types that owls select for hunting (Castañeda et al., 2021; Wendt & Johnson, 2017), a result that is corroborated here. Nest box occupancy before and after fire was positively correlated with amount of grassland and riparian lands, as well as amount of fire edge, and negatively correlated with amount of forest (Huysman & Johnson, 2021). Though the patterns were weaker in the RUF results, owls also appeared to preferentially hunt in these same land cover types. Similarly to previous work, we found that owls showed some statistical avoidance of vineyard at the individual level both before and after the fires, though 29% of all observed recorded locations were in vineyards, likely because of the amount of vineyard land available in this study area (Castañeda et al., 2021). Our finding that Barn Owls in our system generally use land cover types in proportion to their availability, with some selection for uncultivated land cover types at the individual level, suggests that owls prefer open, uncultivated lands, but are likely to remove rodents from vineyards if that is the most accessible land cover type. Due to the hierarchical nature of habitat selection (Kristan, 2006; Mayor et al., 2009), birds may choose nest sites and then foraging areas around them, or select foraging areas first and then the nest sites within them (Lawler & Edwards, 2006). Variance‐decomposition analysis has shown that Barn Owls choose their nest site location primarily based on the characteristics of the landscape at the home range scale (Wendt & Johnson, 2017), but nuances around this issue remain unresolved in our study system. Since home range foraging habitat availability plays a large role in nest site selection, it is understandable that many of the same land cover variables associated with nest box occupancy are also associated with hunting habitat utilization.

Barn Owls showed resilience in their selection of land cover types after disturbance, which is likely related to the adaptability and historically wide distribution of the species. Barn Owls are rodent predators, but they are opportunistic when rodent availability changes, so their adaptability may have buffered their response against landscape changes (Hindmarch et al., 2012; Kross et al., 2016; Tores et al., 2005). This adaptability and opportunistic behavior are qualities that have led Barn Owls to succeed in diverse landscapes throughout the world (Roulin, 2020). Additionally, Napa Valley has a history of frequent fires dating back 10,000 years or more to the use of fire as a management tool by Indigenous peoples—the Mishewal Wappo, Napa, Caymus, Canijolmano, and Mayacama (Grossinger, 2012). Since Barn Owls are native to this region, there are evolutionary reasons to believe that the species would be resilient and able to take advantage of landscape changes caused by wildfire. Yet, recent fires are different from the frequent, low‐intensity fires that were utilized by Indigenous groups, with potential ecological effects (Jones et al., 2020; Keeley & Brennan, 2012; Steel et al., 2015). The combination of warming, increased fine fuels, and other climatic factors are causing the fires of the western United States to be more severe than the fires of the past (Gillson et al., 2019). As the western United States, as well as other fire‐prone regions throughout the world, see increased wildfire severity (Rogers et al., 2020), it is increasingly important to understand whether native species such as Barn Owls are resilient to changing fire regimes.

These results suggest a short‐term resilience to wildfire, but it is unknown if the fires had any long‐term effects on fitness and prey availability that could not be detected through occupancy and telemetry monitoring. Rodent monitoring, diet studies, and the combination of diet and telemetry data could help to determine where pest rodents are most available and from where they are actually removed (Johnson & St. George, 2020). It is also possible that Barn Owls that nested near recently burned areas had different nest success from those that nested away from the fire, so analysis of reproductive success could reveal more short‐term and long‐term effects of fires on this population. Some effect of fire on reproduction is likely, given previous research showing that landscape features can influence nest success (Charter et al., 2010; Hindmarch et al., 2014). Furthermore, our conclusions are limited based only on the GPS tracking of females in our system. Though our methods ensured that we tracked individuals during maximum hunting effort to feed nestlings, we do not know how this behavior differs between males and females. Recent work with Barn Owls in Switzerland showed that males have smaller home ranges than females, but the two sexes used land cover types similarly (Séchaud et al., 2021). As technology advances and it becomes more feasible to track males which weigh less than females, it will be a priority to track both sexes to understand their habitat use in our study system. Moreover, our study design involved data collection before and after a naturally occurring fire; it was not a fully controlled experiment with all tagged individuals randomly assigned to fire and tracked both before and after its effects, which limits the strength of inference. The conclusion that fires did not have a significant short‐term impact on Barn Owl habitat selection suggests a resilience to wildfire, but more work is needed to determine long‐term effects and Barn Owls' full potential for pest control in years with and without disturbance.

This study reveals that Barn Owls are resilient to drastic landscape changes caused by wildfires, a finding that is especially significant in California where the threat of severe wildfire is growing (Batllori et al., 2013; Westerling et al., 2006). Barn Owls made opportunistic use of recently burned areas in both nesting (Huysman & Johnson, 2021) and hunting habitat selection, but their use of land cover types was not noticeably different as a result of the fires. Considering the known importance of landscape composition for the delivery of pest control (Lindell et al., 2018; Tscharntke et al., 2005), the ability of Barn Owls to use the landscape in a similar way before and after fire is an encouraging result for wine producers hoping to use Barn Owls as rodent pest control. (TableA1).

## CONFLICT OF INTEREST

The authors declare no competing interests.

## AUTHOR CONTRIBUTION

**Allison E. Huysman:** Conceptualization (equal); Data curation (lead); Formal analysis (lead); Investigation (equal); Methodology (equal); Project administration (equal); Writing – original draft (lead); Writing – review & editing (equal). **Matthew D. Johnson:** Conceptualization (equal); Data curation (supporting); Formal analysis (supporting); Funding acquisition (lead); Investigation (equal); Methodology (equal); Project administration (equal); Writing – original draft (supporting); Writing – review & editing (equal).

## Data Availability

The datasets generated during and analyzed in this study are available in the Movebank Data Repository, https://doi.org/10.5441/001/1.82b5h1rk (Huysman et al., 2021).

## APPENDIX 1

**TABLE A1:** ∆AICc scores for two ANOVA models where response variable is the distance between the centroid of the utilization distribution and either the nest box or the fire perimeter, calculated for all 32 birds

Response variable	Year * Burn	Year + Burn	Year	Burn	Null
Distance to nest box^a^	3.37	2.89	0.28	2.32	**0.00**
Distance to fire perimeter^b^	2.09	0.45	55.48	**0.00**	58.38

Note

*year* represents pre or post fire and *burn* indicates whether that nest box ever experienced fire. Model with lowest AICc score for each ANOVA is bolded. If the wildfires strongly affected habitat selection, then top models should include additive or interactive models with year and burn as important predictors.

^a^ Lowest AICc = 520.86.

^b^ Lowest AICc = 504.57.
